# Impact of late gadolinium enhancement image acquisition resolution on neural network based automatic scar segmentation

**DOI:** 10.1016/j.jocmr.2024.101031

**Published:** 2024-03-01

**Authors:** Tobias Hoh, Isabel Margolis, Jonathan Weine, Thomas Joyce, Robert Manka, Miriam Weisskopf, Nikola Cesarovic, Maximilian Fuetterer, Sebastian Kozerke

**Affiliations:** a Institute for Biomedical Engineering, University and ETH Zurich, Zurich, Switzerland; b Institute of Diagnostic and Interventional Radiology, University Hospital Zurich, University of Zurich, Zurich, Switzerland; c Department of Cardiology, University Heart Center, University Hospital Zurich, University of Zurich, Zurich, Switzerland; d Center of Surgical Research, University Hospital Zurich, University of Zurich, Zurich, Switzerland; e Department of Health Sciences and Technology, ETH Zurich, Zurich, Switzerland; f Department of Cardiothoracic and Vascular Surgery, German Heart Center Berlin, Berlin, Germany

**Keywords:** Cardiovascular magnetic resonance, LGE imaging, Scar quantification, Neural networks, Deep learning, Automatic segmentation

## Abstract

**Background:**

Automatic myocardial scar segmentation from late gadolinium enhancement (LGE) images using neural networks promises an alternative to time-consuming and observer-dependent semi-automatic approaches. However, alterations in data acquisition, reconstruction as well as post-processing may compromise network performance. The objective of the present work was to systematically assess network performance degradation due to a mismatch of point-spread function between training and testing data.

**Methods:**

Thirty-six high-resolution (0.7×0.7×2.0 mm^3^) LGE k-space datasets were acquired post-mortem in porcine models of myocardial infarction. The in-plane point-spread function and hence in-plane resolution Δx was retrospectively degraded using k-space lowpass filtering, while field-of-view and matrix size were kept constant. Manual segmentation of the left ventricle (LV) and healthy remote myocardium was performed to quantify location and area (% of myocardium) of scar by thresholding (≥ SD5 above remote). Three standard U-Nets were trained on training resolutions Δx_train_ = 0.7, 1.2 and 1.7 mm to predict endo- and epicardial borders of LV myocardium and scar. The scar prediction of the three networks for varying test resolutions (Δx_test_ = 0.7 to 1.7 mm) was compared against the reference SD5 thresholding at 0.7 mm. Finally, a fourth network trained on a combination of resolutions (Δx_train_ = 0.7 to 1.7 mm) was tested.

**Results:**

The prediction of relative scar areas showed the highest precision when the resolution of the test data was identical to or close to the resolution used during training. The median fractional scar errors and precisions (IQR) from networks trained and tested on the same resolution were 0.0 percentage points (p.p.) (1.24 - 1.45), and − 0.5 - 0.0 p.p. (2.00 – 3.25) for networks trained and tested on the most differing resolutions, respectively. Deploying the network trained on multiple resolutions resulted in reduced resolution dependency with median scar errors and IQRs of 0.0 p.p. (1.24 – 1.69) for all investigated test resolutions.

**Conclusion:**

A mismatch of the imaging point-spread function between training and test data can lead to degradation of scar segmentation when using current U-Net architectures as demonstrated on LGE porcine myocardial infarction data. Training networks on multi-resolution data can alleviate the resolution dependency.

## 1 Introduction

Myocardial scar mass derived from cardiovascular magnetic resonance (CMR) late gadolinium enhancement (LGE) imaging [1] is considered the gold standard for non-invasive myocardial viability assessment [2] in the context of acute and chronic myocardial infarction (MI) [3], [4]. LGE imaging can identify reversible myocardial dysfunction before coronary revascularization [5]. Further, myocardial scar mass is of prognostic value in patients with ischemic and non-ischemic cardiomyopathies [6], [7], [8], [9], [10], [11], [12], [13], [14], [15].

In clinical practice, myocardial scar mass quantification requires segmentation of epicardial and endocardial contours (epi- and endo-contours) of the left ventricle (LV) in LGE short-axis view images. With limited contrast, precise delineation of myocardial borders can be challenging, even when anatomical information from aligned functional cine imaging is at hand.

Scar tissue segmentation is either conducted manually or using semi-automatic thresholding methods. For the latter, the two recommended methods are thresholding at 50% of the maximum hyperenhanced myocardial signal intensity, referred to as full-width-at-half-maximum (FWHM), or thresholding at n times the standard deviation (n-SD) of mean healthy myocardial signal [2], [16]. Both thresholding methods require manual segmentation of epi- and endo-contours followed by delineation of hyperintense (FWHM) or remote tissue (n-SD), respectively [16], [17]. Comparisons of the manual and the two semi-automatic approaches suggest no significant difference in segmented scar volumes and reproducibility [11], [15], [17], [18], [19]. Generally, manual segmentation is time-consuming and requires well-trained observers as well as standardized criteria to account for variations in CMR sequences and hardware. In a multi-center study, significant interobserver differences in %LV mass were reported [20], indicating limited generalization of the classification of scar data.

Several attempts to limit human interactions in scar segmentation have been proposed. These include semi- and fully automatic methods, either combining co-registration of cine and LGE images [21], [22] or only LGE images in combination with prior knowledge on constraints and inter-slice smoothness [19], [23]. Karim et al. provide a review and comparison of eight algorithms on a multicenter, multivendor LGE CMR database for atrial scar segmentation. Methods include thresholding, region-growing, graph-cuts, active contours, expectation-maximization, and k-means [24]. A more recent review of fibrosis and scar segmentation is given in [25] including deep learning-based methods. Convolutional neural networks (CNN) have been used for myocardial scar segmentation [26], especially as contributions to segmentation challenges [27]. Moccia et al. proposed a semi-automatic method, where LV contours are manually traced by an expert and then a fully convolutional neural network is trained to segment scar. This approach outperformed the version where the scar was directly segmented on LGE images [28].

Given that myocardial borders in LGE CMR images are often difficult to identify, many approaches fuse information from different sequences. Fahmy et al. proposed to use both cine and LGE images to improve the robustness and accuracy of LGE scar quantification [29]. The availability of publicly available datasets which include LGE, T2-weighted, and cine images (e.g. the MyoPS 2020 dataset [30]) have fostered the development of fused segmentation models. Cui et al. employed curriculum learning, where images are cropped to different sizes [31]. The network is trained with gradually increasing image sizes, making it increasingly more challenging to identify the LV. Wang et al. used deep supervision, where each layer contributes to the loss function [32]. The weight of each contribution is optimized as a hyperparameter using reinforcement learning. Numerous approaches incorporate adjacent slice information or prior information through cascaded pipelines. For example, Fahmy et al. trained a 3D CNN with a sliding window that processes three slices at a time [12]. The final segmentation is achieved by aggregating the individual results per slice. Cui et al. trained a two-stage model, where the first network segments the LV and blood pool, and the second network segments the scar and edema regions [35]. Lustermans et al. implemented a cascaded pipeline with LV bounding box prediction, myocardium prediction, and scar segmentation [36]. Segmentation performance was further enhanced by training augmentation with synthetic images. Finally, Popescu et al. implemented ACSNet, which identifies the region-of-interest, then segments the scar, and as a third step, a network ensures anatomical accuracy [37]. Datasets from various imaging centres, vendors, and multiple CMR sequences with a range of spatial resolutions (1.5–2.4×1.5–2.4×6–8 mm^3^) were used.

The standard data processing approach for CNN based segmentation includes resampling or interpolation of image data to obtain training and test data with constant field-of-view (FOV) and matrix size, i.e. constant apparent in-plane resolution, as well as normalized contrast [12], [29], [33], [34], [37]. However, varying data processing has shown to produce biased results [38], [39]. Of note, the effective image resolution may differ from the apparent resolution as defined by the ratio of FOV and matrix size. The imaging point-spread function (PSF) depends on various factors including sequence and reconstruction parameters as well as post-processing steps before image-domain data is saved as DICOM files for further processing. Accordingly, the impact of changes of the effective image resolution, while FOV and matrix size remain constant, need to be studied relative to a reference. In absence of in-vivo ground-truth data, post-mortem high-resolution data obtained from animal models may be used as reference to study accuracy of scar segmentation methods [40], [41].

The objective of the present study was to investigate the impact of varying effective image resolutions on automatic LGE myocardial scar quantification using a representative U-Net-type network [42]. To generate reference segmentations for LGE data, high-resolution data from post-mortem porcine MI models (N = 36) are used; training and test data of different in-plane resolutions are derived from the reference k-space data. Four different networks are trained at in-plane image resolutions of Δx_train_ = 0.7, 1.2, 1.7 mm, and a mix of resolutions ranging from Δx_train_ = 0.7 to 1.7 mm and tested on resolutions ranging from Δx_test_ = 0.7 to 1.7 mm. The network predictions are compared against the n-SD thresholding approach with n = 5, i.e. SD5, using manual LV and remote region annotations, based on signed fractional errors of scar mass, interquartile ranges and Dice scores.

## 2 Methods

### 2.1 Animal cohort and handling

Thirty-six domestic healthy female swine (Sus scrofa domestica, breed: Swiss large white, body weights (BW) 60–85 kg were used for the experiments. All swine were fully anesthetized and intubated for positive pressure ventilation. General anesthesia was maintained with isoflurane (2–3%) in 100% oxygen. Heparin was administered intravenously and repeated every hour to maintain an activated clotting time of > 250 s.

**Fig. 1:**
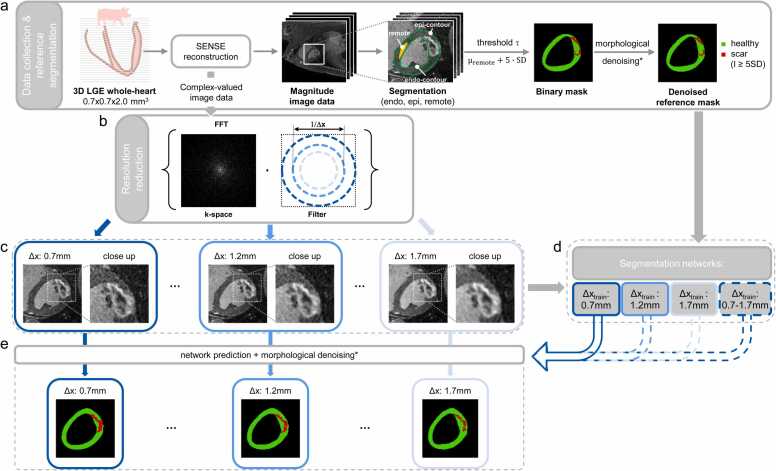
Processing of images and reference (REF) segmentation. (a) Late gadolinium enhancement (LGE) image acquisition and SENSE reconstruction with manual expert observer segmentation of epi- and endo-contours as well as healthy remote myocardium. Semiautomatic reference scar segmentation using SD5 thresholding at acquisition resolution is followed by morphological denoising of scar masks. Healthy myocardium and dense scar (I > SD5) are shown in green and red, respectively. Resolution reduction by multiplication of k-space data with a low-pass filter, while keeping field-of-view and matrix size constant. (c) Examples of resulting images with in-plane resolutions Δx_train_ = 0.7 mm, 1.2 mm and 1.7 mm, respectively. (d) Four segmentation networks are trained for the given resolutions. (e) Exemplary segmentation mask predictions for the network trained at Δx_train_ = 0.7 mm (solid blue arrow) including identical (*) morphological denoising. SENSE, sensitivity encoding; SD: standard deviation

Acute MI and microvascular obstruction in the apical septum, or basal inferior and lateral wall was induced by 90 min occlusion of the left anterior descending coronary artery (LAD) or the left circumflex artery, respectively. After the procedure and subsequent imaging, performed in the acute phase of MI (6 h after reperfusion), all animals were euthanized in deep anesthesia by lethal injection of pentobarbital. All animal handling, procedures and protocols were approved by the Cantonal Veterinary Office (Zurich, Switzerland). The data processing pipeline is illustrated in Fig. 1.

### 2.2 Data acquisition and reconstruction

Imaging was conducted on a clinical 1.5 T Philips Achieva MR system (Philips Healthcare, Best, The Netherlands), delivering 40 mT/m at 200 T/m/s, and using a 5- or 32-channel cardiac receiver array. Functional as well as gadolinium-enhanced imaging was performed as part of a standard ischemic heart disease protocol [43].

A gadolinium based contrast agent (Gadobutrol, Gadovist, Bayer Healthcare, Berlin, Germany) was injected (0.2 mmol/kg BW). The animal was euthanized 20 min after contrast administration and LGE imaging was immediately performed post-mortem and in-situ. High-resolution images were acquired using a sequence consisting of a 3D inversion-recovery gradient-echo sequence with the following parameters: TE/TR 2.3/4.7 ms, acquisition resolution 0.7×0.7×2.0 mm^3^, parallel imaging sensitivity encoding (SENSE) reduction factor: 2.2, and flip angle: 15°. The optimal inversion delay was estimated using an inversion delay scout sequence, maximizing signal contrast between affected and remote myocardium (∼290 ms). Total acquisition time of one 3D dataset was 25 min. Images were reconstructed from undersampled k-space data using SENSE [44]. Coil sensitivities were derived from a pre-scan including both surface and body coil data using code available in MRecon (GyroTools LLC, Zurich, Switzerland) to ensure constant intensity level appearance. The 3D whole-heart image data was resliced in short-axis orientation yielding 2D slices (∼60–80 per dataset) covering the LV from apex to base.

### 2.3 Data post-processing

The high-resolution images were analyzed using GTVolume (GyroTools LLC, Zurich, Switzerland). Apical and basal slices were omitted when endocardial borders were not distinguishable without referring to functional imaging, or the extent of muscle around the blood pool was smaller than 100%. In all other slices, epi- and endocardial borders were manually delineated. Healthy remote tissue regions were labelled by drawing contours around myocardium that showed no hyperenhancement. The papillary muscles were excluded for the analysis. Images and segmentation masks were exported and further processed using Python (version 3.8).

### 2.4 Reduction of image resolution

To test the dependence on image resolution for training and prediction, the high-resolution data was projected to resolutions of Δx $\in \left\{0.7,\ldots ,1.7\right\}\;$mm, with the highest resolution corresponding to the original acquisition resolution of Δx_orig_∼0.7mm. To this end, the reconstructed complex-valued image data was Fourier transformed to k-space, multiplied with a 2D Gaussian lowpass filter and inverse Fourier transformed back to image space. The effective image resolution was defined as the full-width-at-half-maximum of the PSF resulting from the inverse Fourier transform of the 2D Gaussian lowpass filter. Accordingly, a constant matrix size of (128×128) at a constant FOV was kept throughout. An outline of the resolution reduction is shown in Fig. 1.

### 2.5 Reference masks

Masks for myocardial- and scar-tissue were generated using the n-SD approach with n = 5, i.e. by assigning the threshold τ according to

(1) $$\tau =\mu_{\mathrm{remote}}+5\cdot \sigma_{\mathrm{remote}}$$

where $\mu_{\mathrm{remote}}$ and $\sigma_{\mathrm{remote}}$ denote the mean signal amplitude and its standard deviation (SD) in the remote healthy tissue, respectively. The reference masks were calculated for the original acquisition resolution (Δx_orig_∼0.7mm) and used for all other image resolutions. Class associations are presented as red-green (RG) images where colour channels correspond to healthy myocardium in green and scar (I>SD5) in red. To avoid mislabelling of single pixels as scar in the high-resolution data, the reference (REF) masks were denoised by applying morphological opening to the binary scar mask. To ensure inclusion of individual pixels adjacent to the scar clusters, a dilation operation was performed on the total scar mask before it was multiplied with the original mask. An example case of this morphological denoising operation with the original and the denoised mask is shown in Fig. 1.

### 2.6 Network training

A five-fold cross-validation scheme was applied to increase statistical meaning of the reported errors. In each cross-validation pass the total dataset of 36 subsets was split into training (1585 - 1717 slices) and test data (332 - 464 slices), where the data subset splits were performed volume-wise. For each of the three in-plane resolutions: Δx = 0.7 mm, 1.2 mm, and 1.7 mm, a U-Net style architecture network was trained using the ‘Segmentation Models for PyTorch’ library [45]. For the encoder path, a ResNet34 [46] with pretrained weights from the ImageNet dataset (www.image-net.org) was used to accelerate convergence and increase accuracy [47], [48], [49]. The number of downsampling steps in the encoder path was set to 5, and the number of convolutional channels in the decoder path were (256, 128, 64, 32, 16). In the decoder path, batch norm operations were used after every convolution. As optimizer, Adam with an initial learning rate of 1e^−3^, $\beta_{1}=0.9$ and $\beta_{2}=0.999$ was employed. The training batch size was set to 16 and as loss function the multi-class Dice loss was used. One-hundred epochs were used to train the network.

Before passing the LGE images to the network, the images were cropped to a 128 × 128 matrix centered at the LV. This process matches the procedure of other work [29], [34], [37]. Furthermore, the test image intensity was normalized to [0,1] per image. Data augmentation was applied during training, which randomly transformed the images in each epoch. The transformations included elastic deformations with a random deformation grid, intensity transformations, and added Gaussian noise. The reference masks were transformed accordingly for geometric transformations. Test time augmentation was used i.e. test data was augmented 8-fold using multiple 90-degree rotations combined with horizontal or vertical flipping. At test/validation time, the mask prediction for each test image was obtained by applying the same 8 image augmentation operations and by computing the mean of all predictions after reversing the augmentation.

In addition to the networks trained at Δx_train_ = 0.7 mm, 1.2 mm, and 1.7 mm, a fourth network was trained with multiple resolutions. The same network architecture was used as described above. For each training sample, a random resolution was chosen from the set of test resolutions {0.7 mm, 0.9 mm, 1.1 mm, 1.2 mm, 1.3 mm, 1.5 mm, 1.7 mm}. The predicted segmentations were post-processed using the same morphological operations as for the reference masks to avoid isolated pixels being classified as scar.

### 2.7 Error metrics

Predictions of each network (trained at Δx_train_ = 0.7, 1.2 and 1.7 mm) were evaluated on the test data with resolutions ranging from Δx_test_ = 0.7 to 1.7 mm. The resulting estimation of myocardium and scar tissue area was compared pairwise against the reference myocardial masks and 5-SD scar segmentation (SD5), respectively.

For all resolutions Δx, the areas (in mm^2^) of the tissue segmentations ($Ƭ\in \left\{\mathrm{MYO},\mathrm{SD}5\right\}$) per slice with pixels $\tilde{p}$ of mask M is given as:

(2) $$A_{P}(Ƭ)={\Delta x}^{2}\;\Sigma_{\overset{\sim }{p}\in M}(P\left(\overset{\sim }{p}\right)\wedge Ƭ)$$

where P is the segmentation derived from either network prediction or reference (REF) at the highest resolution.

The signed relative fractional error in percent for myocardial tissue is then derived:

(3) $$\Delta \mathrm{MYO}=\frac{{A_{\mathrm{network}}\left(\mathrm{MYO}\right)-A}_{\mathrm{REF}}(\mathrm{MYO})}{A_{\mathrm{REF}}(\mathrm{MYO})}$$

For the scar tissue, the signed pairwise fractional error in percentage points (p.p.) is derived as:

(4) $$\Delta \mathrm{SCAR}=\frac{A_{\mathrm{network}}(\mathrm{SCAR})}{A_{\mathrm{REF}}(\mathrm{MYO})}-\frac{A_{\mathrm{REF}}(\mathrm{SCAR})}{A_{\mathrm{REF}}(\mathrm{MYO})}.$$

To provide full descriptive statistics, resulting marginal distributions of fractional errors with their medians and interquartile range (IQR) are reported. Network precision is indicated by IQR.

To capture the spatial correspondence of network segmentation and thresholding the Sorensen-Dice coefficient was calculated between tissue masks as area of overlap divided by the total number of pixels in both masks [50]:

(5) $$\mathrm{Dice score}(Ƭ)=2\cdot \frac{\Sigma_{\overset{\sim }{p}}\{(\mathrm{network}\left(\overset{\sim }{p}\right)\wedge Ƭ)\cdot (\mathrm{REF}\left(\overset{\sim }{p}\right)\wedge Ƭ)\}}{\Sigma_{\overset{\sim }{p}}\{(\mathrm{network}\left(\overset{\sim }{p}\right)\wedge Ƭ)+(\mathrm{REF}\left(\overset{\sim }{p}\right)\wedge Ƭ)\}}.$$

Dice score marginal distributions with their medians and IQR are derived. For all three training resolutions, Dice score histograms at the training resolutions are derived, respectively. To enhance readability, Dice score histograms are visualized as their kernel density estimations for all investigated test image resolutions. When both SD5 thresholding and network indicated no scar, the Dice score was set equal to 1.

## 3 Results

### 3.1 Cohort data

Over the 36 subjects (2049 slices), mean (± SD) myocardial area was 1136 mm^2^ (± 349 mm^2^) on REF, and the relative area of scar (i.e. SD5) was 5% (± 9%) of the myocardial area. Scar extended up to 53% of the myocardial area in few subjects (∼10%). The corresponding marginal distributions are shown in Supporting Information Fig. S1 in Additional File 1.

### 3.2 Resolution dependency of network performance

**Fig. 2:**
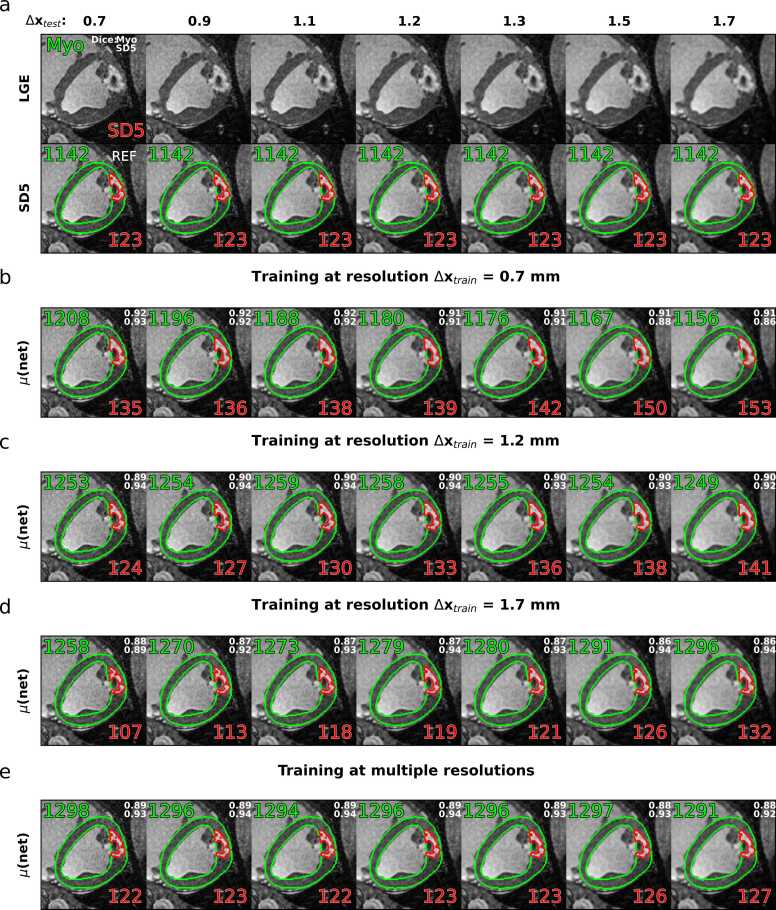
Example late-gadolinium-enhancement (LGE) dataset. Images with varying in-plane resolution Δx and reference SD5 thresholding segmentation masks are shown in (a). Corresponding predictions using networks trained on these resolutions are shown in (b-d), respectively. Predictions for a network trained on mixed resolutions from Δx_train_ = 0.7 mm to 1.7 mm are shown in (e). Healthy myocardium is shown in green and scar (SD5) in red. Regional areas in mm^2^ for myocardium and scar are given as numbers in green and red, respectively. Dice scores relative to SD5 thresholding on REF are given in the top right corner of the shown frames. REF, reference; SD, standard deviation

Fig. 2 shows an example case with an infarct in the anterior and anterolateral myocardial wall. Resolution reduction leads to partial volume effects and change of noise level in the images, as also shown in the corresponding close-ups in Fig. 1. The reference relative scar area, derived by SD5 thresholding, is 10.8% in this case. Network prediction when using high-resolution training, Δx_train_ = 0.7 mm (Fig. 2b), overestimated scar areas in all cases. Compared to the reference, scar areas are overestimated by 9.8% for the highest test resolution and 24.4% for the lowest test resolution data, respectively. At training resolution Δx_train_ = 1.2 mm (Fig. 2c), predicted scar areas are overestimated by 0.8% for the highest test resolution and 14.6% for the lowest test resolution, respectively. At lowest training resolution, Δx_train_ = 1.7 mm (Fig. 2d), the predicted scar areas are underestimated by 13.0% for the highest test resolution and overestimated by 7.3% for the lowest test resolution. The network trained on a combination of resolutions in the range Δx_train_ = 0.7 – 1.7 mm resulted in scar areas underestimated by 0.8% for the highest test resolution and overestimated by 3.3% for the lowest test resolution, exhibiting the most robust results across all test resolutions.

**Fig. 3:**
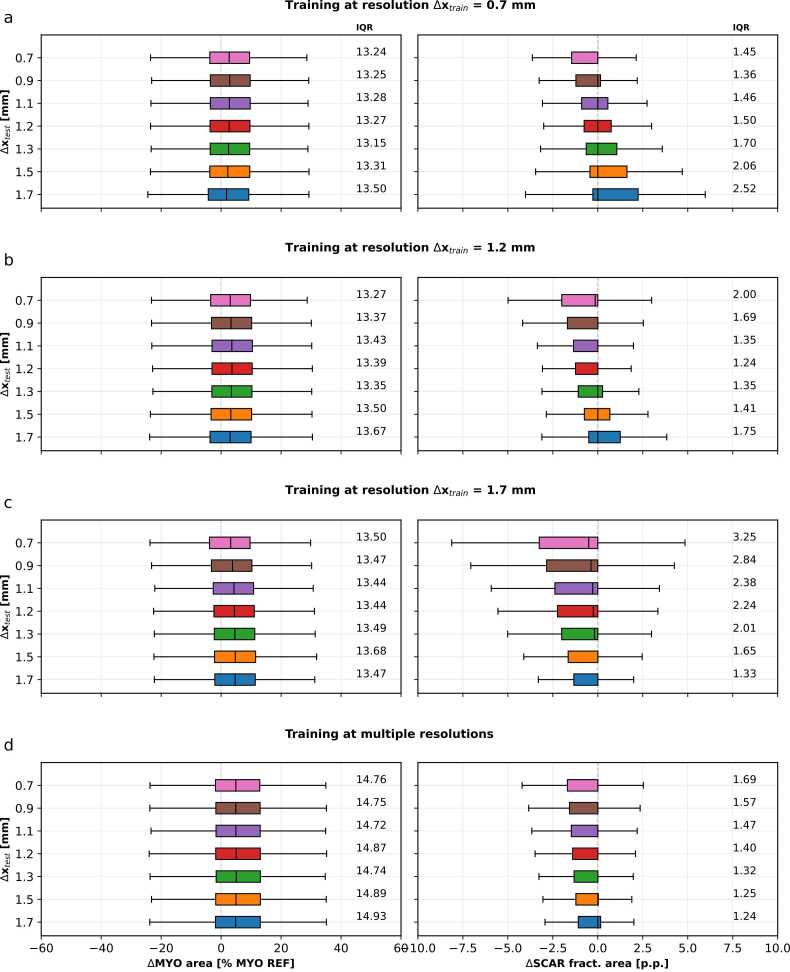
Boxplot analysis of signed errors between network predictions and SD5 thresholding as a function of in-plane resolutions Δx_test_ from 0.7 mm to 1.7 mm is shown for networks trained on Δx_train_ = 0.7 mm (a), 1.2 mm (b) and 1.7 mm (c), and multiple resolutions Δx_train_ = 0.7 mm to 1.7 mm (d). Left and right columns show boxplots for myocardium (MYO) and scar (SCAR) predictions, respectively. Interquartile ranges (IQR), which indicate network precision, are given in the legend. SD, standard deviation

Results for fractional scar area are shown in Fig. 3. Marginal distributions of fractional errors show that scar areas are consistently underestimated at higher test resolutions and overestimated at lower test resolutions.

At the highest training resolution, Δx_train_ = 0.7 mm (Fig. 3a), the median of ΔMYO is between 1.8% and 2.9%. The network tends to overestimate the myocardial area for all investigated test resolutions. The IQR lies between 13.15 and 13.50, indicating the highest precision for Δx_test_ = 1.3 mm. Median scar errors (ΔSCAR) are constant at 0.0 p.p. across all test resolutions. Fig. 3a shows a trend where the scar area is increasingly overestimated, or less underestimated, for lower-resolution images. The IQR lies between 1.36 and 2.52, with the highest precision for Δx_test_ = 0.9 mm, and the lowest precision for Δx_test_ = 1.7 mm.

For the training resolution Δx_train_ = 1.2 mm (Fig. 3b), the median of ΔMYO is between 3.0% and 3.6%. The network tends to overestimate the myocardial area for all investigated test resolutions. The IQR lies between 13.27 and 13.67, indicating the highest precision for Δx_test_ = 0.7 mm. Median ΔSCAR results are constant at 0.0p.p. for Δx_test_ ≥ 0.9 mm and − 0.15p.p. for Δx_test_ = 0.7 mm. Fig. 3b shows that the scar area is increasingly overestimated (or less underestimated) for lower-resolution images. The IQR lies between 1.24 and 2.00, with the highest precision for Δx_test_ = 1.2 mm and the lowest precision for Δx_test_ = 0.7 mm.

At the lowest training resolution, Δx_train_ = 1.7 mm (Fig. 3c), the median of ΔMYO is between 3.2% and 4.7%. The network tends to overestimate the myocardial area for all investigated test resolutions. The IQR lies between 13.44 and 13.68, indicating the highest precision for Δx_test_ = 1.1 - 1.2 mm. Median ΔSCAR results increase from − 0.5p.p. for Δx_test_ = 0.7 mm to 0.0p.p. for Δx_test_ = 1.7 mm. Fig. 3c shows a trend towards increasingly underestimation of scar area for higher-resolution images. The IQR lies between 1.33 and 3.25, with the highest precision for Δx_test_ = 1.7 mm and the lowest precision for Δx_test_ = 0.7 mm.

The network trained on a combination of resolutions (Fig. 3d) shows relatively constant ΔMYO and ΔSCAR errors across the investigated test resolutions. The median of ΔMYO is between 5.5% and 5.8%. The IQR lies between 14.72 and 14.93, indicating the highest precision for Δx_test_ = 1.1 mm. Median ΔSCAR results remain constant at 0.0p.p. for all investigated test resolutions. The IQR lies between 1.24 and 1.69 with the highest precision for Δx_test_ = 1.7 mm and the lowest precision for Δx_test_ = 0.7 mm.

**Fig. 4:**
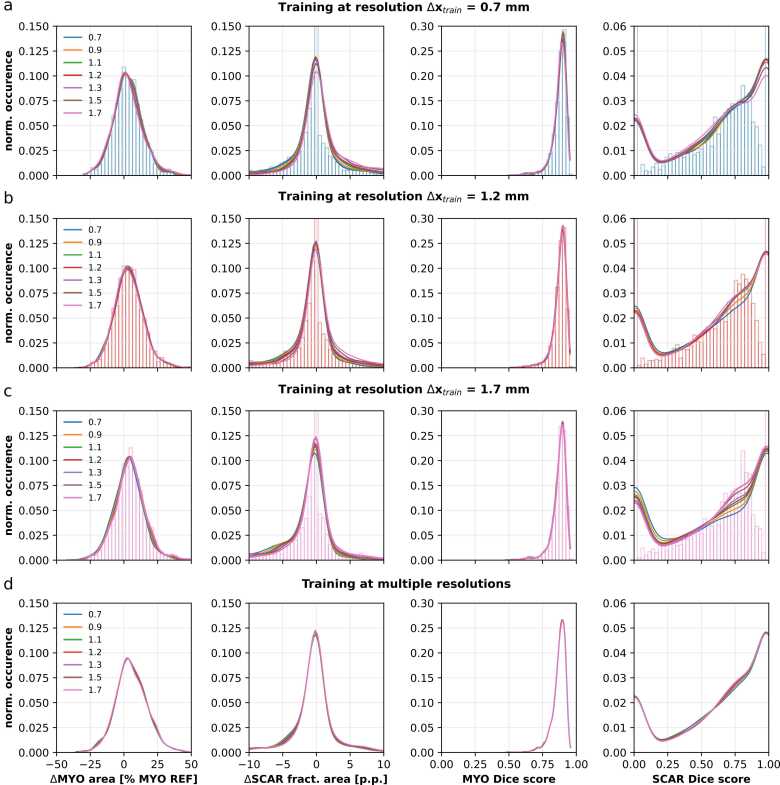
Signed errors and Dice score marginal distributions between network predictions and SD5 thresholding as a function of in-plane resolutions Δx_test_ = 0.7 mm to 1.7 mm are shown for networks trained on Δx_train_ = 0.7 mm (a), 1.2 mm (b), 1.7 mm, and multiple resolutions Δx_train_ = 0.7 mm to 1.7 mm (d). The two left panels show the signed errors as kernel density estimations of histograms for myocardium (MYO) and scar (SCAR), respectively. The two right panels show the Dice scores as kernel density estimations of histograms for MYO and SCAR, respectively. Actual histogram reflects distribution of signed errors and Dice scores at training data resolution.

These results are further reflected in the first two columns in Fig. 4, where marginal distributions of the fractional errors for the myocardium and the scar are shown, respectively. The distributions exhibit a similar shape, peaking at around zero, for all test resolutions.

### 3.3 Spatial correspondence

**Fig. 5:**
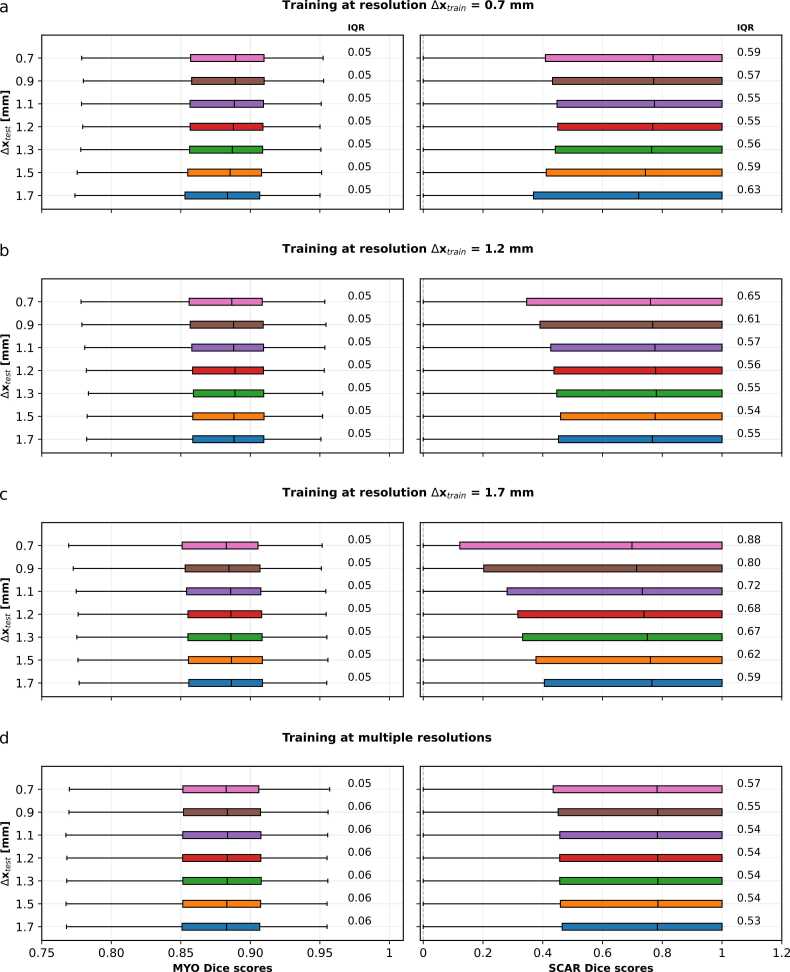
Boxplot analysis of Dice scores between network predictions and SD5 thresholding as a function of in-plane resolutions Δx_test_= 0.7 mm to 1.7 mm are shown for networks trained at Δx_train_ = 0.7 mm (a), 1.2 mm (b), 1.7 mm (c) and mixed multiple resolutions Δx_train_ = 0.7 mm to 1.7 mm (d). Left and right columns show boxplots for myocardium (MYO) and scar (SCAR) predictions, respectively. Interquartile ranges (IQR), which indicate network precision, are given in the legend. SD, standard deviation

Fig. 5 illustrates the results of Dice score comparison (for mean ± SD and p-values see Table 1 in the Additional File 1).

For high-resolution training, Δx_train_ = 0.7 mm (Fig. 5a), median myocardium Dice scores are in the range of 0.88 and 0.89 with a constant IQR of 0.05. The median Dice scores for scar are in the range of 0.72 for Δx_test_ = 1.7 mm and 0.77 for Δx_test_ = 0.7 mm. The IQR lies between 0.55 and 0.63, with the highest network precision given for Δx_test_ = 1.1 mm and 1.2 mm.

For training at Δx_train_ = 1.2 mm (Fig. 5b), median myocardium Dice scores are constant at 0.89 with a constant IQR of 0.05. The median Dice scores for scar are in the range of 0.76 for Δx_test_ = 0.7 mm and 0.78 for Δx_test_ = 1.1 mm - 1.5 mm. The IQR lies between 0.54 and 0.65, with the highest precision given for Δx_test_ = 1.5 mm.

For low-resolution training, Δx_train_ = 1.7 mm (Fig. 5c), median myocardial Dice scores are in the range of 0.88 and 0.89 with a constant IQR of 0.05. The median Dice scores for scar are in the range of 0.70 for Δx_test_ = 0.7 mm and 0.78 for Δx_test_ = 1.7 mm. The IQR lies between 0.59 and 0.88, with the highest precision given for Δx_test_ = 1.7 mm.

The network trained on multiple resolutions (Fig. 5d) reveals relatively constant Dice scores across all investigated test resolutions as compared to the other networks. The median myocardial Dice scores are 0.88 with an IQR between 0.05 and 0.06 for all investigated test resolutions. The median Dice scores for scar are in the range of 0.78 and 0.79. The IQR lies between 0.53 and 0.57, with highest precision given for Δx_test_ = 1.7 mm and lowest precision for Δx_test_ = 0.7 mm.

The last two columns in Fig. 4 show the Dice score distributions for both myocardium and scar. The distributions are similar across all investigated test resolutions. The network trained on low-resolution images (Fig. 4c) shows the largest variability in the Dice scores for scar prediction.

Corresponding full marginal distributions for fractional errors and Dice score analysis are provided as Supporting Information Fig. SI 2–9 in the Additional File 1.

**Fig. 6:**
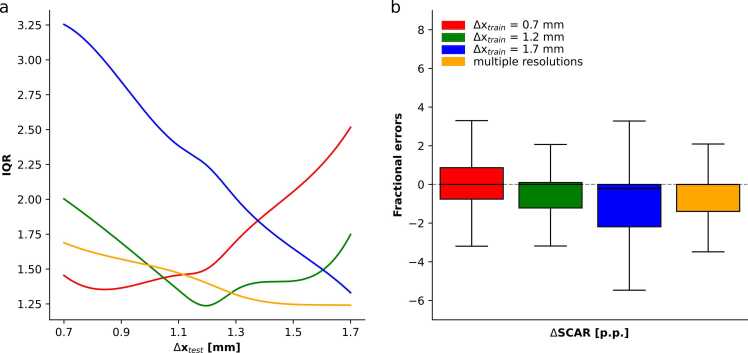
Segmentation performance summary. (a) Interquartile ranges (IQR) of the signed scar errors over all investigated in-plane resolutions Δx_test_ = 0.7 mm to 1.7 mm are shown for networks trained on Δx_train_ = 0.7 mm, 1.2 mm, 1.7 mm, and multiple resolutions. (b) Signed scar (SCAR) errors median and IQR, aggregated across all investigated in plane resolutions Δx_test_ = 0.7 mm to 1.7 mm, are shown for networks trained at Δx_train_ = 0.7 mm, 1.2 mm, 1.7 mm, and multiple resolutions.

Network errors and precisions for scar segmentation are summarized in Fig. 6, where the IQRs of the scar errors for each network and each test resolution are shown. The network trained on high-resolution images (Δx_train_ = 0.7 mm) exhibits a lower IQR, thus higher network precision, for high-resolution test images and decreasing precision for lower resolutions. The network trained at Δx_train_ = 1.2 mm exhibits the best network precision for images of the same resolution and deteriorates for test images of higher or lower resolution. The network trained on low-resolution images (Δx_train_ = 1.7 mm) exhibits a high IQR when applied to high-resolution images, which decreases gradually for lower resolution images. Hence, the network precision is highest on test images of the same resolution as the train images and deteriorates for higher resolution images.

Fig. 6b shows the medians and IQRs of signed fractional errors for scar area averaged over all investigated test resolutions. For training resolutions Δx_train_ = 0.7, 1.2 and 1.7 mm, median (IQR) compares as 0.0 p.p. (1.6 p.p.), 0.0 p.p. (1.3 p.p.) for myocardium, and − 0.2 p.p. (2.2 p.p.) for scar. For the network trained on multiple resolutions, the median (IQR) is 0.0 p.p. (1.4 p.p.).

## 4 Discussion

This work evaluated the impact of changes in the point-spread function and, hence, effective resolution on scar segmentation using standard U-Net type neural networks. The large high-resolution post-mortem dataset allowed network training at different effective resolutions, i.e. enabled a change of the image point-spread function while keeping matrix size and field-of-view constant.

While scar segmentation using post-mortem high-resolution data has previously been shown in studies with pigs (N = 17) and canines (N = 11) [40], [41], the number of cases in these studies would have been too small for the training of networks.

Using a standard U-Net, network-based predictions of relative scar areas showed variability for fractional errors and Dice scores across the investigated test resolutions. All networks underestimated scar areas more often on high-resolution than on low-resolution test images. The networks trained at Δx_train_ = 0.7 mm and 1.2 mm overestimated the scar area more frequently on low-resolution test images. This is partly explained by the fact that reduced resolution corresponds to a wider PSF, which leads to spatial information being smeared out over neighbouring pixels, i.e., spatial signal variations occur on a larger scale and are smoother.

A key challenge of the present work was the derivation of SD5 reference scar masks. As pointed out by Heiberg et al. [51], n-SD thresholding depends on the signal-to-noise ratio (SNR) in the data. Low SNR causes underestimation of the scar area, and high SNR results in overestimation. Given that reference scar masks were derived at the highest available resolution (Δx = 0.7 mm), where the SD5 thresholding is more likely to underestimate the true scar area, the networks trained on low-resolution images learned to segment the scar conservatively. However, when applied to high-resolution images, the networks tended to increasingly underestimate the area compared to the reference. Conversely, networks trained on high-resolution images and applied to low-resolution images tended to overestimate scar area. Accordingly, network precision, indicated by the IQR, where smaller IQR corresponds to higher network precision, was found to depend on effective resolutions as well. Networks deployed on images with the same or similar resolution as in the training exhibited a higher precision than networks applied to images with differing resolutions (Fig. 5a). The precision of the network trained at the lowest resolution (Δx_train_ = 1.7 mm) was most susceptible to changes in test resolutions. When applied to high-resolution images, the IQR increased by 144% compared to the IQR when applied to low-resolution images. Hence, when the effective resolution did not match the resolutions during network training, the network confidence decreased, resulting in segmentations more likely to be inaccurate.

Regarding Dice scores of scar predictions, no significant trend was found for networks trained at Δx_train_ = 0.7 mm and 1.2 mm. However, the network trained on low-resolution images (Δx_train_ = 1.7 mm) showed a trend towards higher Dice scores for lower-resolution images. The median Dice score increased by 0.07 between the network tested on the highest-resolution images and the network tested on the lowest-resolution images. Additionally, the difference in IQR was 0.29, indicating that networks trained on low-resolution images but applied to high-resolution images suffered in segmentation accuracy and precision. The Dice score distributions (Fig. 4) underline this finding as well, where the network trained on low-resolution images exhibited higher resolution-dependent variability. The distributions can be explained by the nature of the dataset and the Dice scores. The Dice score distributions of the scar predictions show that a large number of Dice scores are zero or one. A large number of slices do not contain scars (∼30% of the samples) and thus only have healthy myocardium in the corresponding reference mask. At the same time, the Dice score is not defined for cases that show no scar area in both the reference mask and the prediction. However, because the network correctly identified that no scar was present, a Dice score of 1 was assigned in that case. This happened for the majority (∼90%) of samples that did not contain scars. The network did not predict any scar in ∼13% of all the samples. On the other hand, in ∼4% of all the samples, the network predicted scar, where there was none. This resulted in zero Dice scores (Fig. 4).

Standard U-Nets trained on a single resolution were found to be less robust to changes in the test image resolution when compared to the network trained on multiple different resolutions. This was reflected in the marginal distributions of signed fractional area errors and Dice scores and is in agreement with work by Popescu et al. [37].

Unlike scar predictions, no resolution-dependent trends were found for myocardial segmentation across the investigated test resolutions. The fractional errors were bound to 1.8%. However, all trained networks overestimated myocardial area between 2.9% to 5.8%. This bias is associated with the manual delineation of the myocardium to be too conservative, i.e., endo- and epi-contours drawn by the observer within the myocardium rather than at the interfaces.

### 4.1 Limitations

A limitation of the present work is that the impact of effective resolution on segmentation accuracy was only investigated for a standard U-Net architecture. Other pipelines were not investigated, e.g., multi-modality learning models for LGE LV segmentation incorporating LGE, cine, and atlas data [52], [53].

The mean myocardial and scar Dice scores were on the order of 0.87 – 0.88 and 0.59 – 0.66, respectively. While this is already comparable with established work [26], [27], the model’s capacity to learn high-resolution data has been tested using a deeper encoder (ResNet50) to test the hypothesis that the number of parameters in the network is potentially insufficient for the high-resolution data. Results did not indicate a difference (see Supporting Information Fig. SI 10 in the Additional File 1). Accordingly, the network training was considered sufficient for the described analysis. However, more advanced techniques, such as deep supervision and attention networks, have not been employed [54]. An aspect that has also not been investigated in this work is the through-plane resolution of the 3D image datasets. Future work should include this analysis, also regarding SNR, as this is of further interest when 3D LGE imaging is used, and scar segmentation algorithms are applied [55], [56]. Future work should also generate reference validation epi- and endocardial segmentations from multiple experienced observers to mitigate uncertainties in the current reference segmentation dataset. In addition, manual scar segmentation and thresholding using the FWHM method [57] should be considered to generate alternative reference segmentation, as there is no consensus for the optimal method of quantitative scar assessment [16]. Such data could also serve as a better reference for interobserver variability and overall segmentation accuracy comparisons. Future experiments might further consider testing the trained models using prospective or external datasets with low-resolution data. Of note, differences in timing relative to contrast agent administration and changes in heart shape over time post-mortem in-situ may compromise isolation of resolution-dependent effects. Finally, the PSF-dependent bias and variability demonstrated in our work indicate the importance of considering and reporting acquisition rather than reconstruction resolution as a crucial parameter, as well as SNR, when designing and evaluating scar segmentation networks.

## 5 Conclusion

This study showed that variations in imaging point-spread function affect scar segmentation using standard U-Net architectures. Networks trained and tested on different resolutions suffer from reduced network accuracy and precision as compared to networks trained and tested on the same or similar resolution. Training networks on multi-resolution data can alleviate the resolution dependency.

## Ethics approval and consent to participate

All animal handling, procedures and protocols were approved by the Cantonal Veterinary Office (Zurich, Switzerland) under the license ZH219/2016 and ZH213/2019.

## Consent for publication

Consent for publication was obtained from all authors.

## Funding

This work was supported by Innosuisse - Schweizerische Agentur für Innovationsförderung, Grant/Award Number: 31010.1..

## CRediT authorship contribution statement

**Maximilian Fuetterer:** Conceptualization, Writing – review & editing. **Nikola Cesarovic:** Investigation. **Miriam Weisskopf:** Investigation. **Robert Manka:** Writing – review & editing. **Thomas Joyce:** Methodology, Writing – review & editing. **Jonathan Weine:** Methodology, Writing – review & editing. **Isabel Margolis:** Conceptualization, Formal analysis, Investigation, Methodology, Validation, Writing – original draft, Writing – review & editing, Data curation. **Tobias Hoh:** Conceptualization, Data curation, Formal analysis, Investigation, Methodology, Validation, Writing – original draft, Writing – review & editing. **Sebastian Kozerke:** Conceptualization, Writing – review & editing.

## Declaration of Competing Interest

The authors declare the following financial interests/personal relationships which may be considered as potential competing interests: Not applicable reports financial support was provided by Innosuisse Swiss Innovation Agency. If there are other authors, they declare that they have no known competing financial interests or personal relationships that could have appeared to influence the work reported in this paper.
