# Supplementary material for: Impact of late gadolinium enhancement image acquisition resolution on neural network based automatic scar segmentation
Source: J Cardiovasc Magn Reson. 2024 Mar 1;26(1):101031. doi: 10.1016/j.jocmr.2024.101031 (PMC10981112; doi:10.1016/j.jocmr.2024.101031)
Supplement: Supplementary file 1 — Supplementary material [file mmc1.pdf]

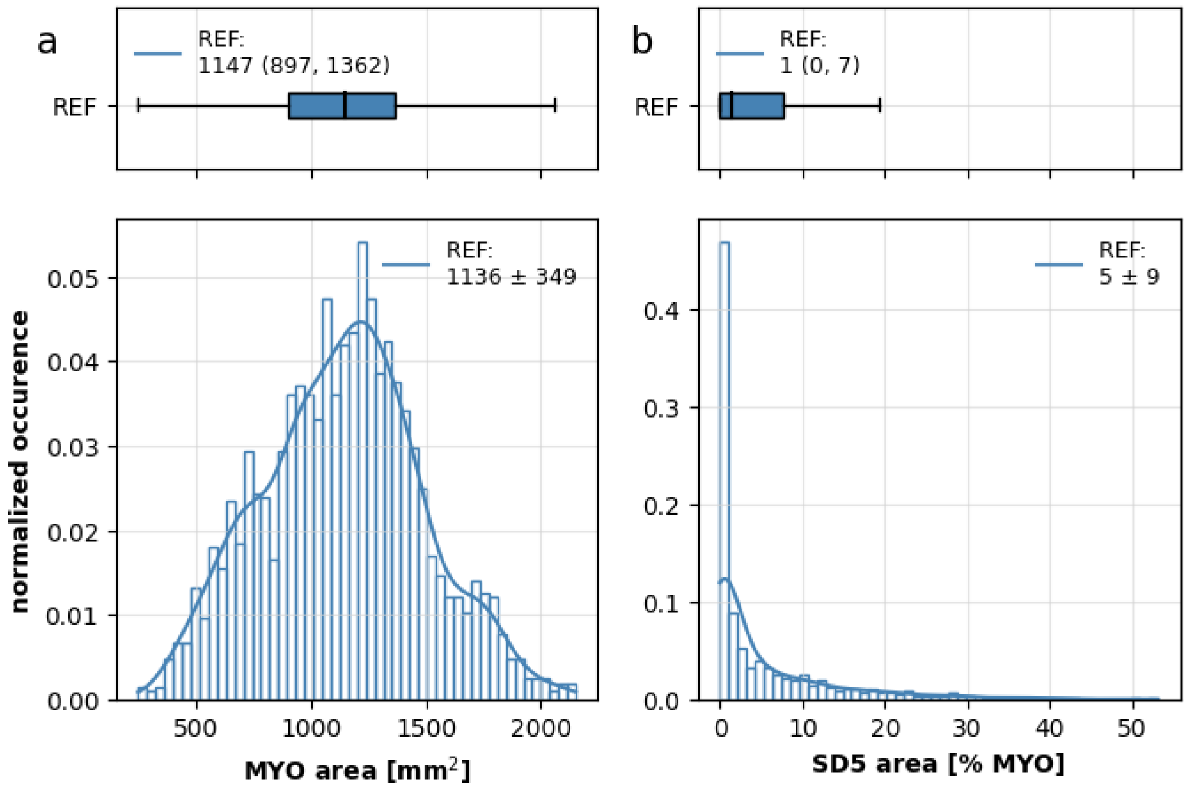

Supporting Information Fig. S1: Cohort data statistics of myocardial (MYO) area in mm and dense scar (SD5) relative areas in %MYO at reference (REF). REF areas are obtained by manual segmentation and n-SD thresholding from data acquired at the highest in-plane resolution Δx = 0.7 mm. Top panels show box-plots of myocardial and relative scar area. Medians (interquartile range minimum, maximum) are given in the legend. Bottom panels show corresponding histograms. To enhance visual appearance, kernel density estimations of distributions are superimposed. Mean ± SDs are given in the legend.

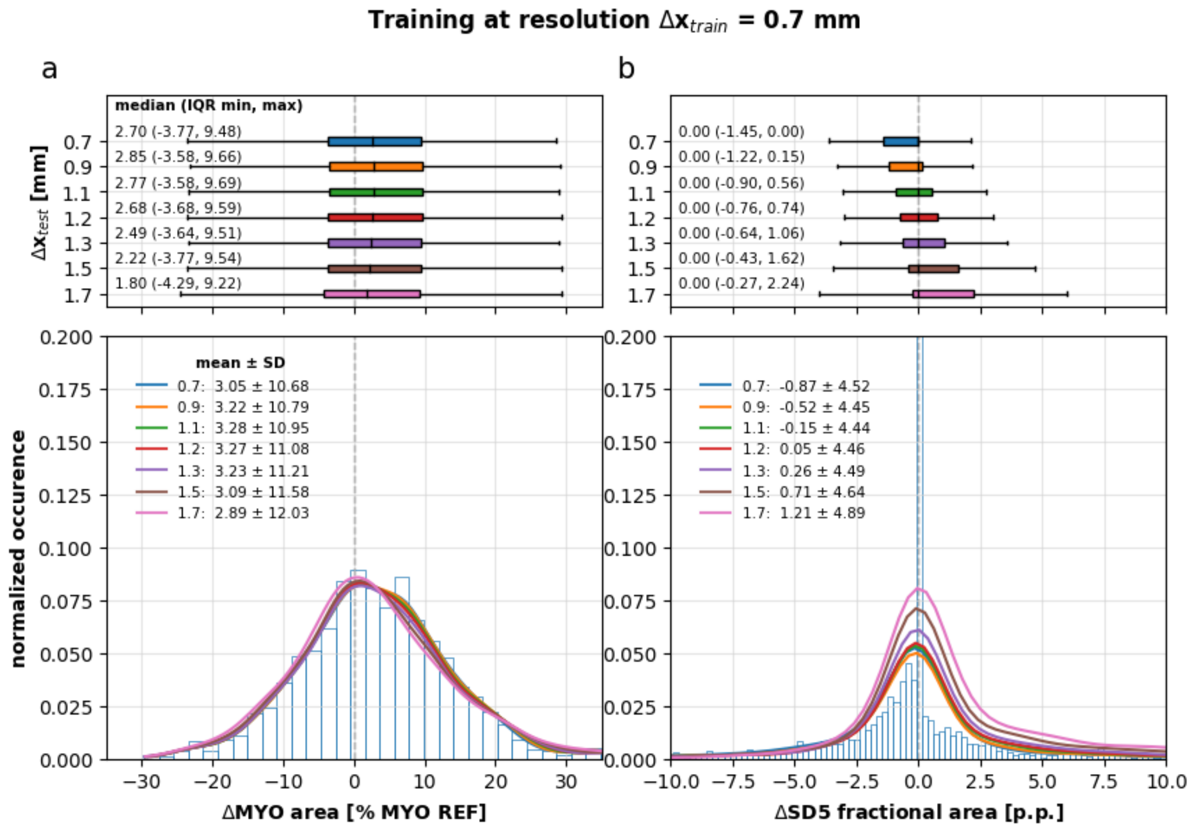

Supporting Information Fig. S2: Marginal distributions of signed errors between network predictions and n-SD thresholding as function of in-plane resolutions Δx_{test} from 0.7 to 1.7 mm are shown for networks trained at Δx_{train} = 0.7 mm. From left to right, panels show signed errors ΔMYO relative to myocardial area at reference and signed fractional errors for dense (SD5) scar, respectively. Top panels show box-plots of the signed error distributions. Medians (interquartile range (IQR) minimum, maximum) are given in the legend. Bottom panels show histograms (blue bars) for the distribution at highest resolution. To enhance visual appearance, kernel density estimations of histograms are superimposed. Mean ± SD are given in the legend. Corresponding data is shown in Figure 3a in the main manuscript.

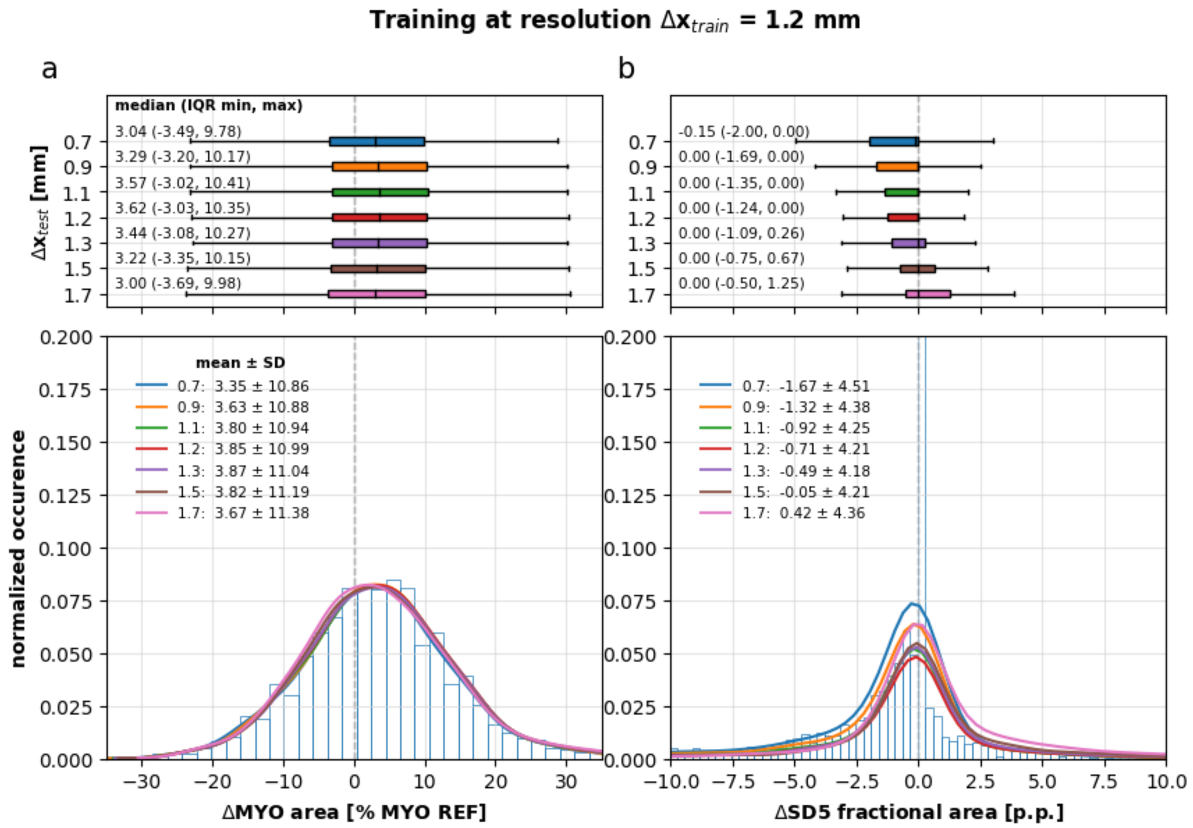

Supporting Information Fig. S3: Marginal distributions of signed errors between network predictions and n-SD thresholding as function of in-plane resolutions Δx_{test} from 0.7 to 1.7 mm are shown for networks trained at Δx_{train} = 1.2 mm. From left to right, panels show signed errors ΔMYO relative to myocardial area at reference and signed fractional errors for dense (SD5) scar, respectively. Top panels show box-plots of the signed error distributions. Medians (interquartile range (IQR) minimum, maximum) are given in the legend. Bottom panels show histograms (blue bars) for the distribution at highest resolution. To enhance visual appearance, kernel density estimations of histograms are superimposed. Mean ± SD are given in the legend. Corresponding data is shown in Figure 3b in the main manuscript.

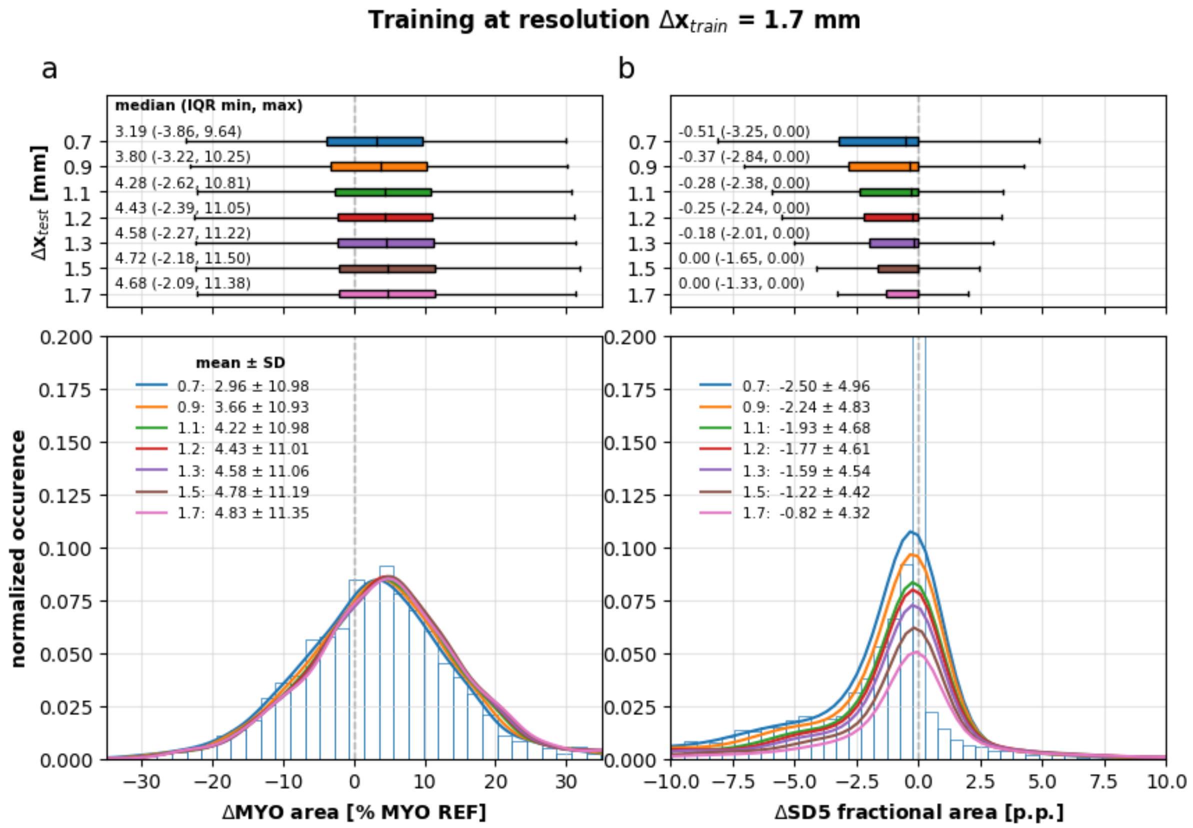

Supporting Information Fig. S4: Marginal distributions of signed errors between network predictions and n-SD thresholding as function of in-plane resolutions Δx_{test} from 0.7 to 1.7 mm are shown for networks trained at Δx_{train} = 1.7 mm. From left to right, panels show signed errors ΔMYO relative to myocardial area at reference and signed fractional errors for dense (SD5) scar, respectively. Top panels show box-plots of the signed error distributions. Medians (interquartile range (IQR) minimum, maximum) are given in the legend. Bottom panels show histograms (blue bars) for the distribution at highest resolution. To enhance visual appearance, kernel density estimations of histograms are superimposed. Mean ± SD are given in the legend. Corresponding data is shown in Figure 3c in the main manuscript.

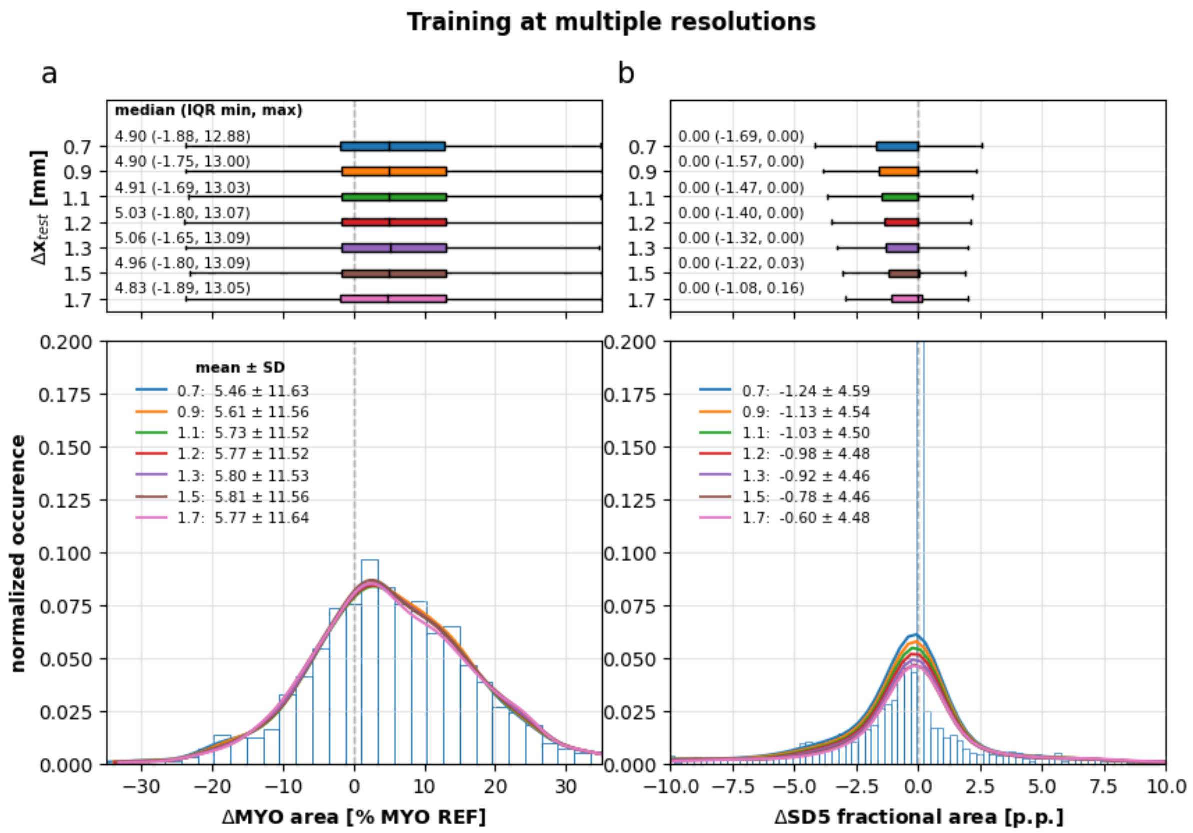

Supporting Information Fig. S5: Marginal distributions of signed errors between network predictions and n-SD thresholding as function of in-plane resolutions Δx_{test} from 0.7 to 1.7 mm are shown for networks trained at mixed Δx_{train} from 0.7 to 1.7 mm. From left to right, panels show signed errors ΔMYO relative to myocardial area at reference and signed fractional errors dense (SD5) scar, respectively. Top panels show box-plots of the signed error distributions. Medians (interquartile range (IQR) minimum, maximum) are given in the legend. Bottom panels show histograms (blue bars) for the distribution at highest resolution. To enhance visual appearance, kernel density estimations of histograms are superimposed. Mean ± SD are given in the legend. Corresponding data is shown in Figure 3d in the main manuscript.

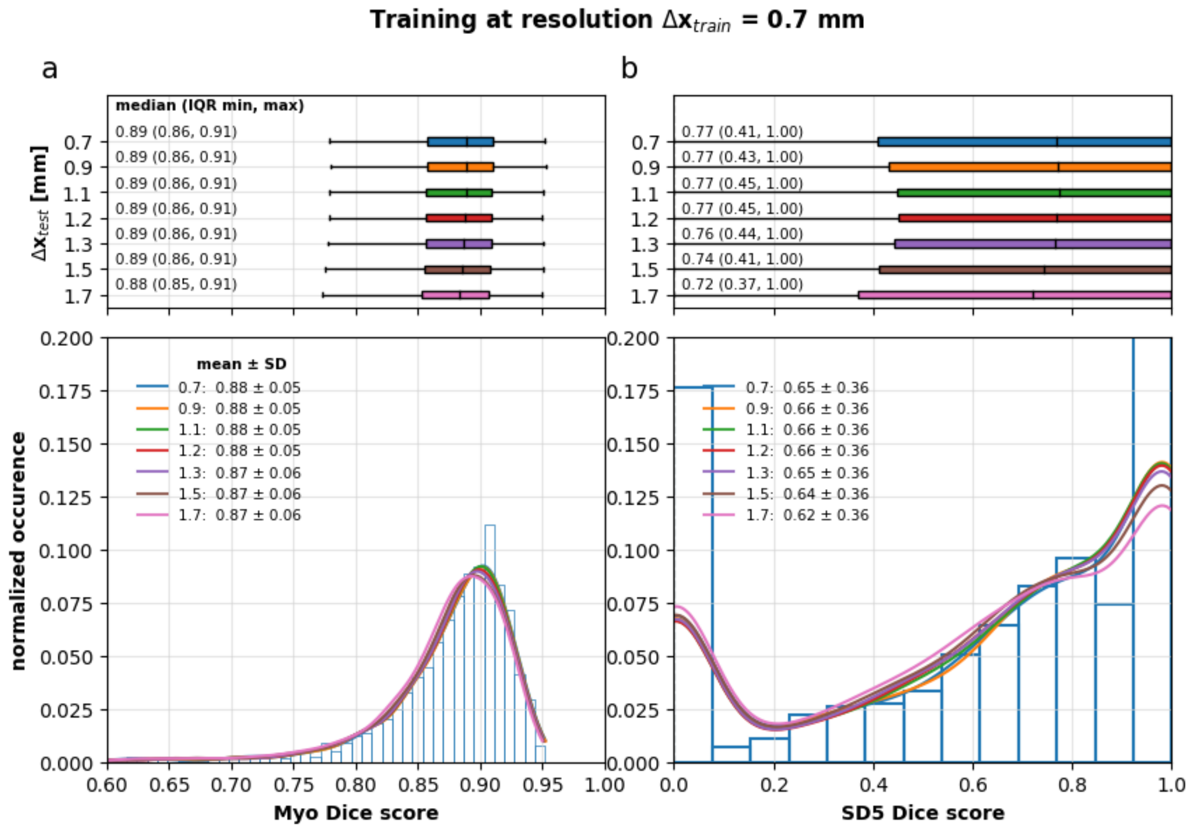

Supporting Information Fig. S6: Dice score marginal distributions between network predictions and n-SD thresholding as function of in-plane resolutions Δx_{test} from 0.7 to 1.7 mm are shown for networks trained at Δx_{train} = 0.7 mm. From left to right, panels show Dice scores for myocardium (MYO) and dense (SD5) scar, respectively. Top panels show box-plots of the Dice score marginal distributions. Medians (interquartile range (IQR) minimum, maximum) are given in the legend. Bottom panels show histograms (blue bars) for the distribution at highest resolution. To enhance visual appearance, kernel density estimations of histograms are superimposed. Mean ± SD are given in the legend. Corresponding data is shown in Figure 4a in the main manuscript.

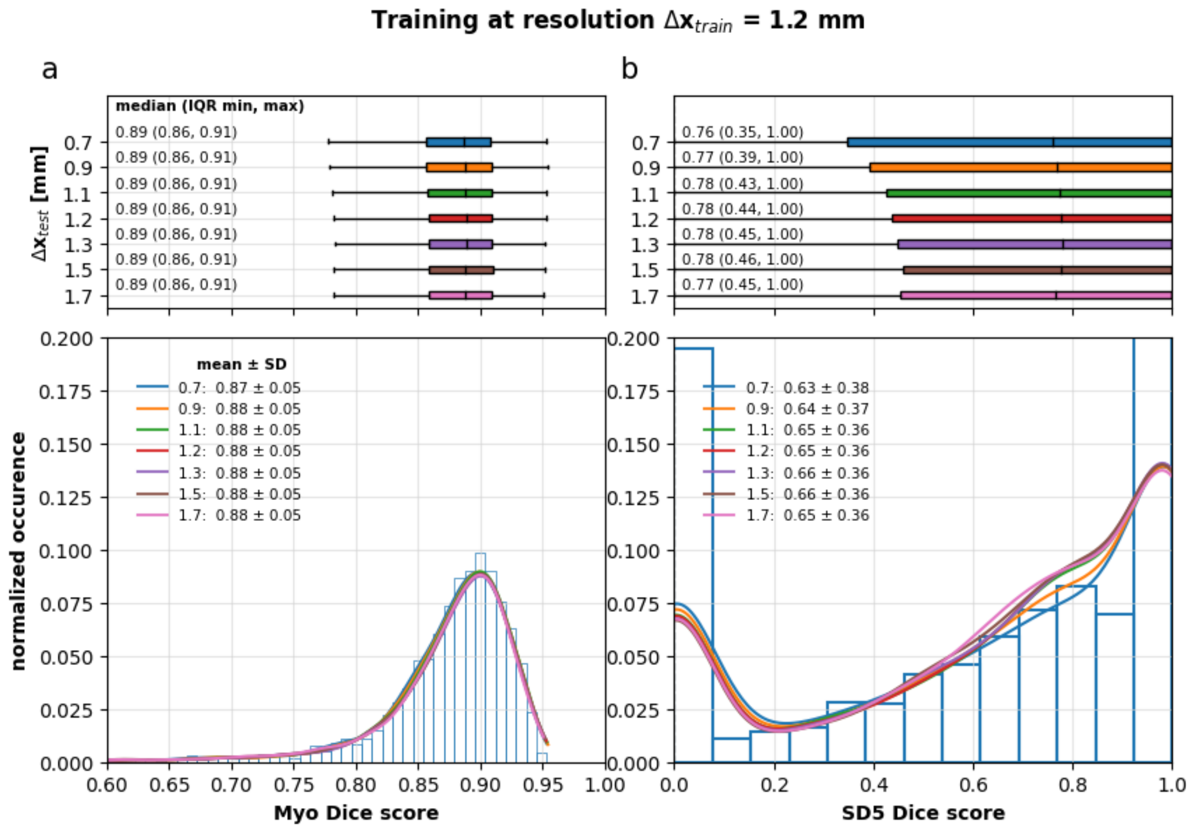

Supporting Information Fig. S7: Dice score marginal distributions between network predictions and n-SD thresholding as function of in-plane resolutions Δx_{test} from 0.7 to 1.7 mm are shown for networks trained at Δx_{train} = 1.2 mm. From left to right, panels show Dice scores for myocardium (MYO), and dense (SD5) scar, respectively. Top panels show box-plots of the Dice score marginal distributions. Medians (interquartile range (IQR) minimum, maximum) are given in the legend. Bottom panels show histograms (blue bars) for the distribution at highest resolution. To enhance visual appearance, kernel density estimations of histograms are superimposed. Mean ± SD are given in the legend. Corresponding data is shown in Figure 4b in the main manuscript.

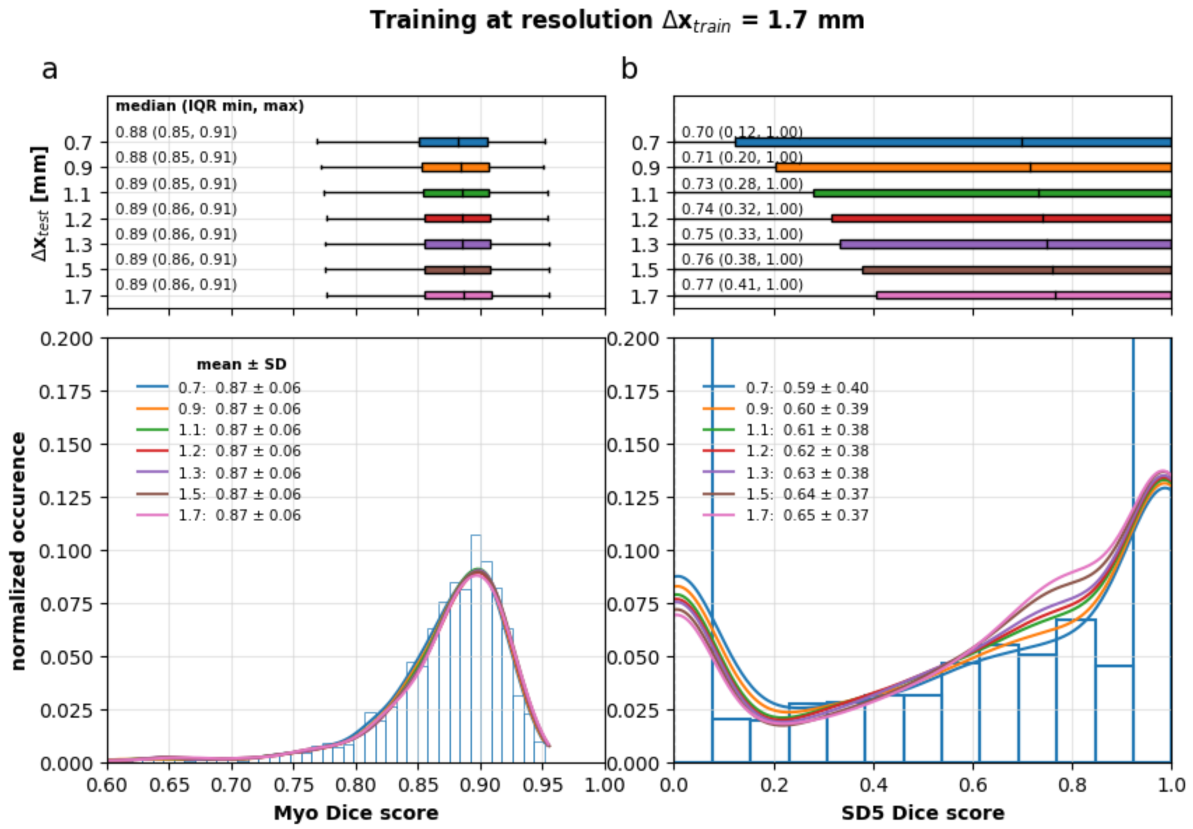

Supporting Information Fig. S8: Dice score marginal distributions between network predictions and n-SD thresholding as function of in-plane resolutions Δx_{test} from 0.7 to 1.7 mm are shown for networks trained at Δx_{train} = 1.7 mm. From left to right, panels show Dice scores for myocardium (MYO) and dense (SD5) scar, respectively. Top panels show box-plots of the Dice score marginal distributions. Medians (interquartile range (IQR) minimum, maximum) are given in the legend. Bottom panels show histograms (blue bars) for the distribution at highest resolution. To enhance visual appearance, kernel density estimations of histograms are superimposed. Mean ± SD are given in the legend. Corresponding data is shown in Figure 4c in the main manuscript.

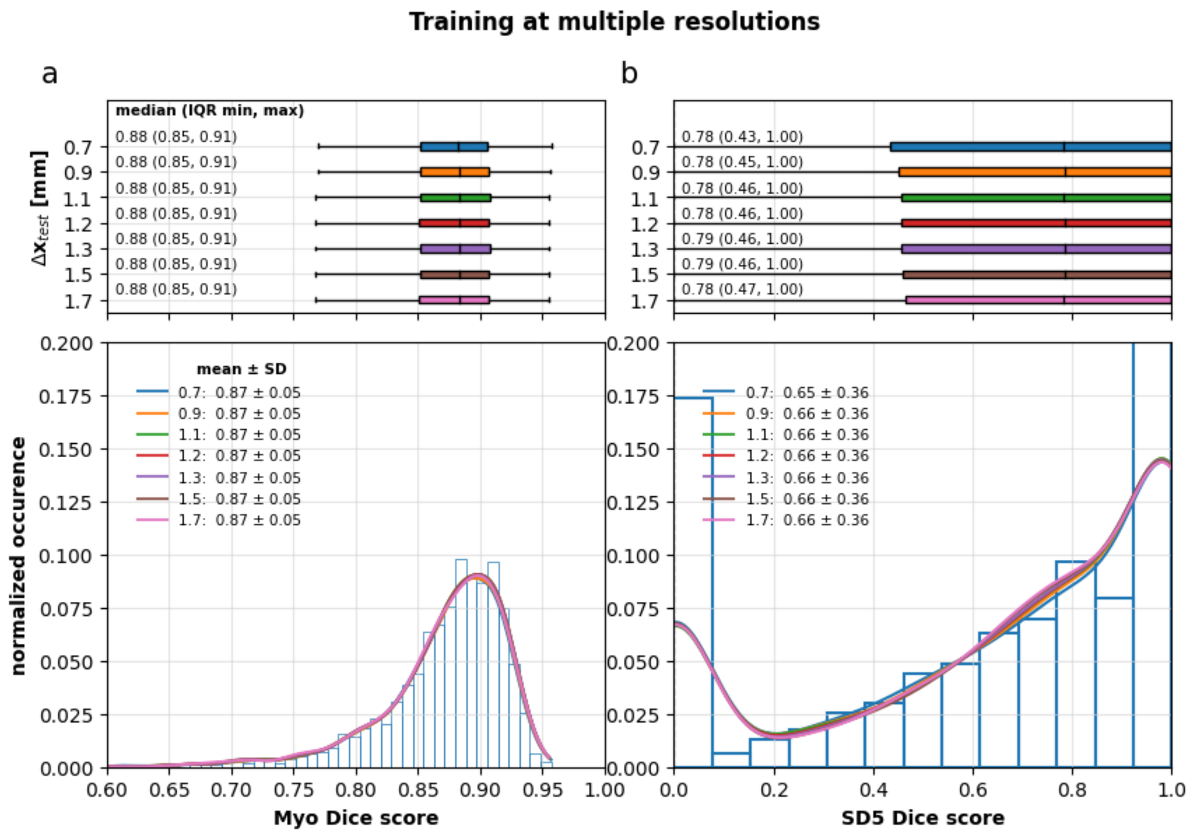

Supporting Information Fig. S9: Dice score marginal distributions between network predictions and n-SD thresholding as function of in-plane resolutions Δx_{test} from 0.7 to 1.7 mm are shown for networks trained at Δx_{train} from 0.7 to 1.7 mm. From left to right, panels show Dice scores for myocardium (MYO) and dense (SD5) scar, respectively. Top panels show box-plots of the Dice score marginal distributions. Medians (interquartile range (IQR) minimum, maximum) are given in the legend. Bottom panels show histograms (blue bars) for the distribution at highest resolution. To enhance visual appearance, kernel density estimations of histograms are superimposed. Mean ± SD are given in the legend. Corresponding data is shown in Figure 4d in the main manuscript.

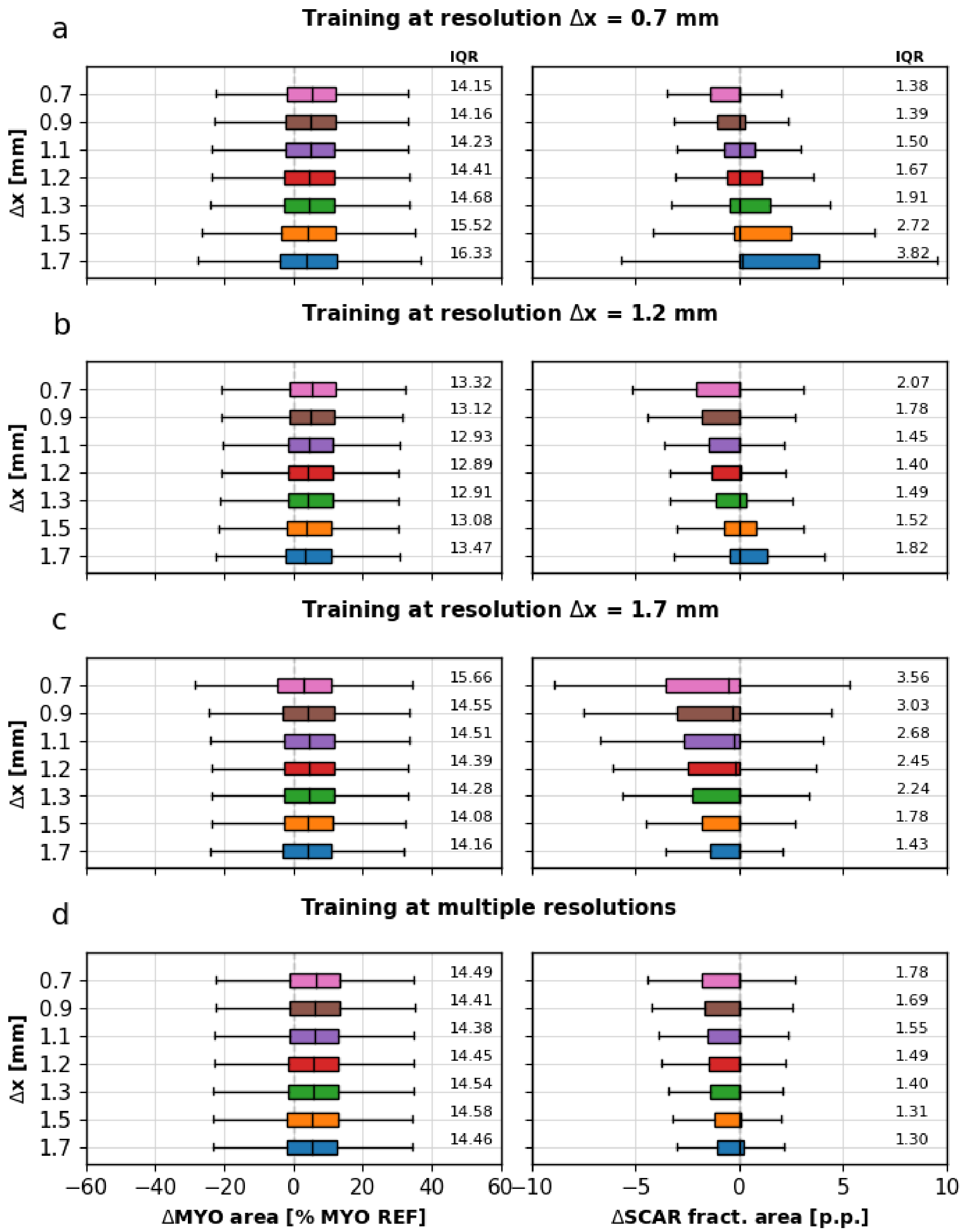

Supporting Information Fig. S10: Boxplot analysis of signed errors between network predictions using a ResNet50 encoder and SD5 thresholding as a function of in-plane resolutions Δxtest from 0.7 mm to 1.7 mm is shown for networks trained on Δx_{train} = 0.7 mm (a), 1.2 mm (b) and 1.7 mm (c), and multiple resolutions Δx_{train} = 0.7 mm to 1.7 mm (d). Left and right columns show boxplots for myocardium (MYO) and scar (SCAR) predictions, respectively. Interquartile ranges (IQR), which indicate network precision, are given in the legend.

Supporting Information Table 1: Dice score results.

Res. Δx_{test}	Training at resolution Δx_{train} = 0.7 mm		Training at resolution Δx_{train} = 1.2 mm		Training at resolution Δx_{train} = 1.7 mm		Training at multiple resolutions	
	Myocardium Dice score	SD5 scar Dice score	Myocardium Dice score	SD5 scar Dice score	Myocardium Dice score	SD5 scar Dice score	Myocardium Dice score	SD5 scar Dice score
**0.7**	0.876 ± 0.054<br>-	0.650 ± 0.365<br>-	0.875 ± 0.053<br>***	0.633 ± 0.378<br>***	0.870 ± 0.047<br>***	0.586 ± 0.401<br>***	0.871 ± 0.053<br>-	0.654 ± 0.364<br>-
**0.9**	0.876 ± 0.054	0.655 ± 0.362<br>***	0.876 ± 0.053<br>***	0.643 ± 0.370<br>***	0.872 ± 0.056<br>***	0.600 ± 0.393<br>***	0.872 ± 0.052<br>-	0.659 ± 0.362<br>-
**1.1**	0.875 ± 0.055<br>***	0.657 ± 0.358<br>**	0.877 ± 0.053<br>***	0.652 ± 0.364<br>***	0.873 ± 0.056	0.614 ± 0.385<br>***	0.872 ± 0.052<br>-	0.661 ± 0.360<br>-
**1.2**	0.875 ± 0.055<br>***	0.656 ± 0.357	0.877 ± 0.053<br>-	0.654 ± 0.363<br>-	0.873 ± 0.056	0.620 ± 0.381<br>***	0.872 ± 0.052<br>-	0.660 ± 0.361<br>-
**1.3**	0.874 ± 0.055<br>***	0.651 ± 0.358	0.877 ± 0.053	0.657 ± 0.360<br>*	0.874 ± 0.055	0.626 ± 0.378<br>***	0.872 ± 0.052<br>-	0.661 ± 0.361<br>-
**1.5**	0.873 ± 0.056<br>***	0.639 ± 0.359<br>**	0.876 ± 0.053<br>**	0.658 ± 0.357	0.874 ± 0.055<br>**	0.639 ± 0.370<br>***	0.872 ± 0.052<br>-	0.662 ± 0.360<br>-
**1.7**	0.871 ± 0.057<br>***	0.619 ± 0.363<br>***	0.876 ± 0.053<br>***	0.654 ± 0.357	0.874 ± 0.055<br>-	0.646 ± 0.365<br>-	0.871 ± 0.053<br>-	0.662 ± 0.359<br>-

Dice score mean ± SD between network predictions and reference n-SD thresholding are shown for networks trained at Δx_{train} = 0.7 mm, 1.2 mm, 1.7 mm and at Δx_{test} ranging from 0.7 to 1.7 mm. P-values of the Wilcoxon signed-rank test between each network and the corresponding reference network (tested at the same resolution as during training) are given: *** $p < 0.01$, ** $p < 0.05$, * $p < 0.1$. Corresponding Dice score marginal distributions and box plots are shown in Fig. 4 and 5, respectively.
